# Mind and body recovery: how rural tourism promotes occupational health in the public health context

**DOI:** 10.3389/fpubh.2026.1829683

**Published:** 2026-07-03

**Authors:** Jinjin Liu, Huanchen Tang, Junjie Li, Yuqi Yang, Miqi Shen, Xiaodong Liu

**Affiliations:** College of Fashion and Design, Donghua University, Shanghai, China

**Keywords:** job crafting intention, perceived restorative environment, physiological recovery, psychological recovery, rural tourism

## Abstract

**Introduction:**

Rising workplace stress has intensified scholarly attention to the relationship between leisure experiences and work-related outcomes. Rural tourism, characterized by restorative environmental qualities, may be closely associated with individuals' psychological and physiological recovery as well as their occupational attitudes.

**Methods:**

Grounded in the Stimulus–Organism–Response (S-O-R) framework, this study developed and tested a structural model linking perceived restorative environmental characteristics of rural tourism to psychological and physiological recovery and subsequently to job crafting intention. Data were collected from 405 employed individuals through a questionnaire survey conducted between September and December 2025. A two-stage analytical approach integrating structural equation modeling (SEM) and artificial neural networks (ANN) was employed.

**Results:**

SEM results indicated that distance from stressors, visual authenticity, experience continuity, and personal compatibility were all significantly and positively associated with job crafting intention, with psychological and physiological recovery operating as parallel mediators. Experience continuity exhibited the strongest overall association, and the pathway through psychological recovery was slightly stronger than that through physiological recovery. ANN analysis further revealed that, within an overall predictive framework, distance from stressors and physiological recovery ranked higher in relative importance, suggesting that restorative mechanisms may involve both linear and nonlinear structures. In addition, tourist behavior patterns moderated several structural paths, indicating that more actively engaged experiences may strengthen the association between restorative perceptions and work-related attitudes. These findings provide empirical support for the structural linkage between rural tourism experiences and job crafting intention, and extend restorative environment research by integrating explanatory modeling with predicitive importance analysis.

## 1 Introduction

Against the backdrop of intensified urbanization and rapid digital transformation, urban employees are confronting unprecedented structural pressures. Prolonged sedentary office work, high-frequency information processing, continuous performance evaluations, and competition for career advancement collectively place them in a persistent state of elevated cognitive load and compressed time resources (1). Particularly within platform-based and mobile work environments, the boundaries between work and personal life have become increasingly blurred. Fragmented communication and the expectation of being “constantly online” have become normalized, leading to escalating issues of emotional exhaustion, attentional decline, and occupational burnout. For this group, traditional long-duration vacations are often impractical under current constraints. Instead, they show a preference for short-distance experiences characterized by manageable time costs, moderate spatial proximity, and immediate restorative effects. In this context, rural tourism in areas adjacent to metropolitan centers—distinguished by natural environments, low-density spatial configurations, and slower-paced lifestyles—has emerged as a significant setting for psychological restoration and emotional regulation among urban workers. Unlike mass sightseeing tourism, this group places greater emphasis on environmental escapability, spatial immersion, and the affective restorative functions of experience rather than mere landscape consumption. Existing research indicates that tourism experiences with restorative qualities contribute to enhanced cognitive flexibility (2), reduced occupational burnout (3), and increased work engagement (4). Therefore, understanding the specific leisure demands shaped by high-pressure occupational structures among urban employees holds important theoretical implications for explaining their rural tourism choice behaviors.

Current scholarship generally follows two lines of inquiry. The first focuses on the influence of rural tourism on tourists' well being, mental health, and behavioral intentions (5, 6), yet its analytical endpoint typically remains within the tourism context, with limited attention to longer-term effects after individuals return to daily life—particularly the workplace. The second line examines the effects of restorative environments on work-related behaviors (7), but research settings are often confined to micro-level interventions such as office spaces, park walks, or virtual environments, emphasizing immediate or short-term outcomes (8, 9). Both streams tend to overlook rural tourism as a more immersive and temporally extended restorative experience that may generate cross-contextual “spillover effects” on individuals' work-related psychological states and behaviors.

Addressing this gap, the present study poses a central question: can the restorative perceptions acquired during rural tourism extend beyond the tourism context and relate to positive behaviors upon returning to work? More specifically, are such experiences associated with heightened job crafting intention—defined as the proactive inclination to adjust work boundaries and optimize occupational cognitions (10)? If rural tourism experiences are linked to this form of intention, the value of tourism may extend beyond stress relief toward potential resource replenishment. However, the internal mechanisms connecting restorative perceptions in tourism to job crafting remain insufficiently clarified. This study proposes that such associations may operate in parallel through physiological and psychological recovery, and therefore advances an integrative explanatory framework to examine these relationships empirically.

To systematically address the aforementioned questions, this study introduces innovations at both the theoretical and methodological levels. Theoretically, drawing upon the Stimulus–Organism–Response (S-O-R) framework, we develop an integrated analytical model linking perceived restorative environmental characteristics (S) to physiological and psychological recovery (O), and subsequently to job crafting intention (R). The model further incorporates tourist behavior patterns as a moderating factor. Methodologically, the study adopts a multi-stage hybrid computational framework: machine learning techniques are first employed to capture unstructured perceptual data, followed by the integration of structural equation modeling (SEM), which provides path-based explanatory power, and artificial neural networks (ANN), which excel in modeling nonlinear relationships. This triangulated methodological approach enhances the robustness of the findings. Specifically, the study addresses three core issues: (1) which rural environmental attributes are associated with physiological and psychological recovery among urban employees; (2) how such recovery may extend into the work domain through dual pathways and relate to job crafting intention; and (3) whether heterogeneity exists across different tourist behavior patterns in this transformation process.

The remainder of this paper is organized as follows. Section 2 reviews relevant literature and develops the research hypotheses and conceptual model. Section 3 describes the research design and analytical methods. Section 4 reports the SEM results. Section 5 presents the supplementary ANN analysis. Section 6 discusses the findings, theoretical contributions, limitations, and directions for future research.

## 2 Literature review and research hypotheses

### 2.1 Job crafting intention

Job Crafting Intention refers to an individual's subjective inclination to proactively adjust task boundaries, relational structures, or the perceived meaning of work in order to enhance the alignment between work and the self (11). Its theoretical foundation lies in job crafting theory, which emphasizes employees' bottom-up (12), self-initiated modifications of job resources and demands to improve performance outcomes and psychological well being. Prior research consistently demonstrates that job crafting behaviors are significantly associated with work engagement, innovative performance, and career adaptability, positioning job crafting as a critical agentic mechanism through which individuals navigate complex work environments (13, 14).

However, under conditions of intensified urbanization and platform-based work systems, the nature of the work context faced by urban employees has undergone profound transformation. High-intensity performance evaluations, persistent competitive pressures, and the norm of being “constantly online” have increasingly rigidified work boundaries, compounded task demands, and placed sustained strain on psychological resources. Within such structural constraints, organizational-level adjustments may offer limited buffering capacity, thereby heightening reliance on individual-level adaptive strategies. Job crafting intention can thus be understood as a preparatory psychological state emerging within high-pressure occupational settings.

For urban workers who operate under prolonged cognitive overload and temporal compression, restorative leisure experiences may represent an important avenue for rebuilding psychological resources and cognitive flexibility. Rural tourism, as an immersive and relatively extended restorative context, provides not only exposure to natural environments and a sense of spatial escape, but may also relate to dual processes of physiological and psychological recovery. These restorative processes may, in turn, be associated with individuals' readiness and capacity to proactively reconfigure aspects of their work. Accordingly, this study incorporates the rural tourism context into the domain of job crafting research, seeking to elucidate how restorative experiences, through psychological and physiological pathways, relate to urban employees' preparatory orientations toward adaptive work behaviors.

### 2.2 The impact of perceived restorative environments on work stress and post-travel behavior

Perceived restorative environment refers to an individual's cognitive and experiential evaluation of the extent to which a particular setting facilitates recovery from psychological fatigue and stress (15). Its theoretical foundation derives primarily from two complementary perspectives. Attention Restoration Theory (ART) posits that environments reduce the depletion of directed attention through “soft fascination,” thereby replenishing cognitive resources and improving psychological functioning. In contrast, Stress Recovery Theory (SRT) emphasizes that natural cues can rapidly elicit positive affect and reduce stress-related physiological arousal, leading to more immediate emotional relief and physiological recovery. These two perspectives correspond respectively to the core mechanisms of psychological and physiological recovery, providing theoretical support for the parallel dual-path model proposed in this study.

Within the ART framework, Kaplan identified four key characteristics of restorative environments: being away, fascination, extent, and compatibility (16). Building on this framework, Hartig et al. developed the Perceived Restorativeness Scale (PRS), establishing the classical four-dimensional measurement structure (17) that has since been adapted and extended across contexts. For example, Laumann et al. (18) differentiated “being away” into geographical distance and psychological escape, forming the Restorative Components Scale (RCS) (18), while White et al. (19) further subdivided “extent” into coherence and scope (19). With the expansion of research into tourism contexts, Lehto et al. (20) emphasized perceived destination restorativeness, advancing the discussion from general natural settings toward a more refined understanding of destination attributes and experiential mechanisms (20).

In tourism research, perceived restorative environment has become a critical bridge linking tourist experiences to post-travel behaviors. Existing studies primarily focus on the “immediate” and “reparative” outcomes of restorative environments, such as emotional improvement and stress reduction, examining how environmental exposure facilitates recovery from prior work-related strain. Regarding post-travel behaviors, the literature largely centers on destination-related consumption intentions, particularly loyalty and word-of-mouth recommendation. Substantial empirical evidence indicates that when tourists perceive strong restorative qualities in a destination, their experience quality, satisfaction, and positive affect increase significantly, which subsequently translate into stronger revisit and recommendation intentions. Nevertheless, although some studies touch upon issues such as work–life balance, whether restorative experiences can stimulate deeper and more forward-looking cognitive and motivational transformations remains insufficiently explored. Specifically, after achieving profound psychophysiological recovery through rural tourism, can this positive experience extend beyond mere stress relief to prompt a reassessment of work roles, career trajectories, and work–life relationships—thereby fostering job crafting intention? This question remains largely unexamined. It is plausible that when individuals feel fully revitalized within restorative environments, they are more capable of breaking free from habitual cognitive patterns, critically evaluating their current work conditions, and developing intentions to adjust their work modes in pursuit of a more fulfilling professional life (21).

### 2.3 Stimulus–organism–response (S-O-R) theory

The Stimulus–Organism–Response (S-O-R) theory was originally proposed by environmental psychologists Mehrabian and Russell in 1974 to explain how environmental stimuli influence individuals' internal psychological states and subsequently shape their observable behaviors (22). The theory posits that environmental stimuli (S) act upon the organism (O), eliciting internal emotional and cognitive responses, which in turn lead to behavioral outcomes (R). In recent years, the S-O-R framework has been widely applied in marketing and consumer behavior research (23), with the conceptualization of both stimulus and organism variables continuously expanding. At the stimulus level, scholars have moved beyond physical environmental cues (e.g., spatial layout, music, and lighting) to incorporate social cues (e.g., service staff attitudes, presence of others) and online environmental cues (e.g., interface design, interactivity, and privacy protection). At the organism level, in addition to emotional states, various psychological and cognitive constructs—such as perceived value, trust, immersion, and psychological distance—have been conceptualized as internal organismic responses. At the response level, research outcomes have evolved from initial indicators such as time spent and purchase behavior to more diverse consequences (24), including satisfaction (25), word-of-mouth intention (26), continuance intention (27), and loyalty (28). A substantial body of empirical evidence, both domestically and internationally, demonstrates that the S-O-R framework effectively elucidates the causal chain linking environmental cues, internal psychological states, and overt behaviors. Accordingly, it has been extensively validated and applied across retail services, tourism and hospitality, online shopping, and social media contexts, providing robust theoretical support for explaining individual behavior from environmental and situational perspectives in the present study.

### 2.4 Research hypotheses

**Figure 1:**
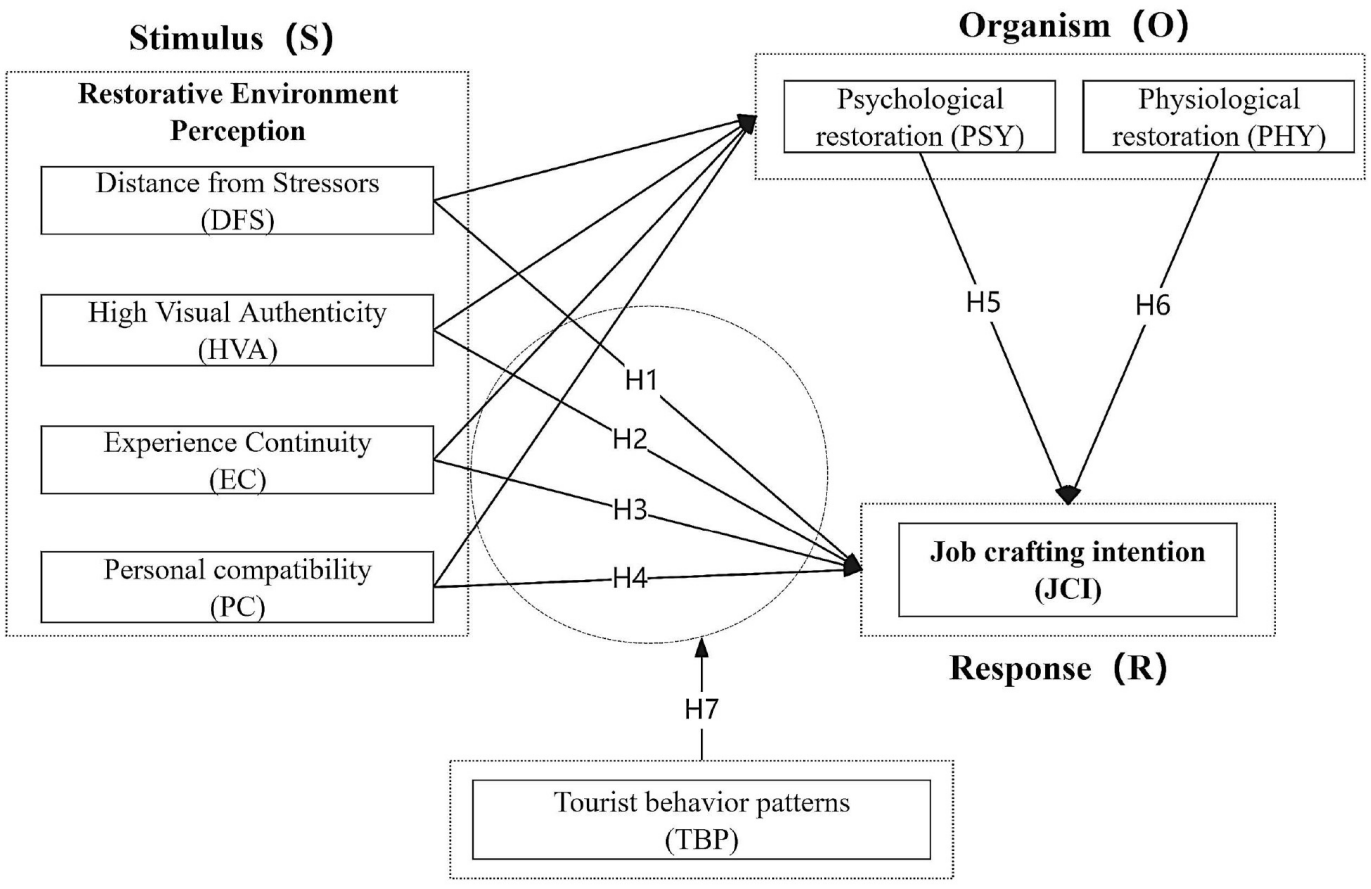
Schematic diagram of the research hypothesis model.

Drawing upon the S-O-R framework, this study develops a core mechanistic model conceptualizing the pathway from perceived restorative environment in rural tourism to psychophysiological recovery and subsequently to job crafting intention. Specifically, the components are defined as follows: Stimulus (S): Refers to perceived restorative environment in rural tourism, namely tourists' overall evaluation of restorative attributes embedded in rural settings, such as fascination, being away, and coherence. This perception constitutes the external situational stimulus influencing individuals' internal states. Organism (O): Encompasses two dimensions—psychological recovery and physiological recovery—representing the internal state changes elicited by exposure to restorative environmental stimuli. These dimensions serve as the central mediating mechanisms linking environmental perception to subsequent behavioral intentions. Response (R): Extends to job crafting intention, defined as tourists' post-travel inclination to proactively adjust and optimize their work roles and patterns as a result of positive psychophysiological changes experienced during tourism. This study aims to examine whether differential pathways exist within the proposed “perceived restorative environment (S) – psychophysiological recovery (O) – job crafting intention (R)” framework, thereby identifying the boundary conditions under which restorative experiences spill over into the work domain. The hypothesized research model is presented in Figure 1.

#### 2.4.1 A four-dimensional conceptualization of perceived restorative environment in rural tourism

Building upon the preceding review of Attention Restoration Theory (ART) and the evolution of its measurement scales, the four restorative characteristics proposed by Kaplan can be contextualized within rural tourism destinations as four key dimensions of environmental perception: experiential continuity, personal compatibility, distance from stressors, and high visual authenticity. Distance from stressors corresponds to ART's concept of being away—psychological and physical detachment from routine demands—and, together with psychological detachment or perceived distance, explains why tourism experiences facilitate recovery and subsequent positive states. High visual authenticity integrates ART's notion of fascination (i.e., soft attention capture) with preferences for natural or authentic cues. Recent tourism research increasingly identifies naturalness, authenticity cues, and destination quality as critical perceptual foundations of restorative experiences (29). Experiential continuity aligns with ART's dimension of extent, referring to environmental coherence, immersion, and the opportunity for sustained exploration. Within tourism contexts, this is often expressed as whether the experience unfolds continuously, with a coherent rhythm and layered progression. Personal compatibility corresponds to ART's concept of compatibility, indicating the congruence between environmental attributes and individuals' goals, preferences, and activity styles. It also reflects the notion of person–place fit, a central construct in restorative environment research. According to ART's fundamental proposition that environments possessing these characteristics facilitate attentional restoration and psychological recovery, this study identifies job crafting intention as the core outcome variable and proposes the following hypotheses:

*H1*: Distance from stressors in rural tourism destinations has a significant positive effect on tourists' job crafting intention.

*H2*: High visual authenticity in rural tourism destinations has a significant positive effect on tourists' job crafting intention.

*H3*: Experiential continuity in rural tourism destinations has a significant positive effect on tourists' job crafting intention.

*H4*: Personal compatibility in rural tourism destinations has a significant positive effect on tourists' job crafting intention.

#### 2.4.2 The parallel mediating roles of psychological and physiological recovery

Grounded in the S-O-R logic, external environmental stimuli—namely, perceived restorative qualities of rural tourism—affect individuals' internal states (psychological and physiological recovery), which subsequently elicit behavioral responses (job crafting intention). This “stimulus–organism–response” chain has been widely applied in tourism restoration research to explain how perceived destination restorativeness influences outcome variables such as well being, destination image, and loyalty (30). Meanwhile, Attention Restoration Theory (ART) and Stress Recovery Theory (SRT) provide more specific theoretical support for the psychological and physiological recovery pathways, respectively. ART emphasizes the replenishment of attentional and cognitive resources, whereas SRT highlights the rapid downregulation of emotional arousal and stress-related physiological activation. Within the present framework, a single-path explanation would be insufficient; rather, psychological and physiological recovery are expected to operate in parallel, jointly transmitting the effects of perceived restorative environment and facilitating the development of job crafting intention. Accordingly, the following hypotheses are proposed:

*H5*: Psychological recovery mediates the relationship between perceived restorative environment and job crafting intention.

*H5a*: Psychological recovery mediates the relationship between distance from stressors and job crafting intention.

*H5b*: Psychological recovery mediates the relationship between high visual authenticity and job crafting intention.

*H5c*: Psychological recovery mediates the relationship between experiential continuity and job crafting intention.

*H5d*: Psychological recovery mediates the relationship between personal compatibility and job crafting intention.

*H6*: Physiological recovery mediates the relationship between perceived restorative environment and job crafting intention.

*H6a*: Physiological recovery mediates the relationship between distance from stressors and job crafting intention.

*H6b*: Physiological recovery mediates the relationship between high visual authenticity and job crafting intention.

*H6c*: Physiological recovery mediates the relationship between experiential continuity and job crafting intention.

*H6d*: Physiological recovery mediates the relationship between personal compatibility and job crafting intention.

#### 2.4.3 The moderating role of visitor behavior patterns

Tourist behavioral patterns refer to differences in how visitors organize their activities and the intensity with which they engage at a destination. Such patterns influence the duration of exposure to environmental cues, the depth of interaction, and levels of physiological arousal, thereby determining whether—and to what extent—perceived restorative environments are translated into recovery outcomes. In natural settings, different types of activities give rise to distinct visitor typologies or activity clusters, leading to differentiated experiences and outcomes (31). From the perspective of restorative environment theory, restorative activities may include both low-arousal static activities (e.g., resting, pausing, sitting or reclining, chatting) and more actively engaged dynamic activities (e.g., walking, ball games, fitness, and recreational exercise). These two categories may operate through different recovery mechanisms: static activities are more likely to facilitate relaxation and psychological detachment, thereby enhancing psychological recovery; dynamic activities, by contrast, are more likely to induce physiological stress reduction and energy activation. Moreover, empirical evidence suggests that simply “sitting and observing” vs. “walking” in natural environments elicits distinct physiological responses (32).

Static behavioral pattern: Primarily characterized by low-intensity activities such as relaxation, reflection, immersion in nature, casual conversation or leisure games, and sitting or standing still. This pattern is more likely to reinforce the pathway of psychological detachment → emotional stabilization → psychological recovery. Dynamic behavioral pattern: Dominated by higher-intensity activities such as engaging in recreational facilities, free play in designated areas, ball sports, walking, or hiking. This pattern is more likely to strengthen the pathway of reduced arousal levels → physiological recovery (33). Integrated (hybrid) behavioral pattern: Involves a combination and alternation between static and dynamic activities, resulting in a more comprehensive experiential structure and richer stimulation. Theoretically, this pattern may simultaneously enhance the efficiency of both psychological and physiological recovery pathways (31). Accordingly, the following hypotheses are proposed:

*H7*: Tourist behavioral patterns moderate the relationship between perceived restorative environment and job crafting intention.

*H7a*: Tourist behavioral patterns moderate the relationship between distance from stressors and job crafting intention.

*H7b*: Tourist behavioral patterns moderate the relationship between high visual authenticity and job crafting intention.

*H7c*: Tourist behavioral patterns moderate the relationship between experiential continuity and job crafting intention.

*H7d*: Tourist behavioral patterns moderate the relationship between personal compatibility and job crafting intention.

## 3 Research design and methods

### 3.1 Research methods and procedure

**Figure 2:**
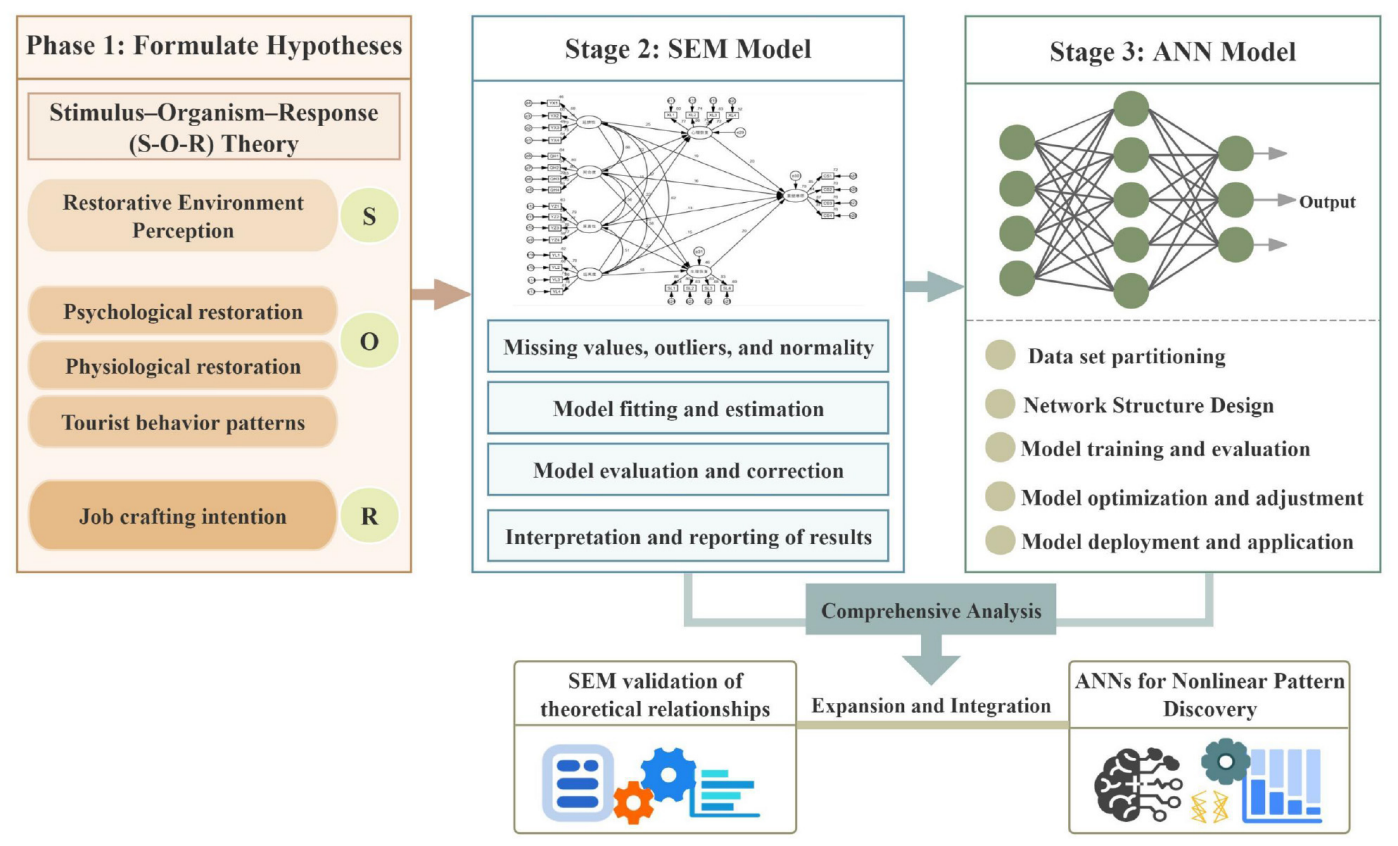
Research methodology pathway diagram.

This study adopts a three-stage analytical framework—qualitative identification, SEM validation, and ANN exploration—to enhance predictive performance and result robustness while maintaining theoretical explanatory power. This integrated approach strengthens the persuasiveness of the findings at both theoretical and practical levels (see Figure 2).

First, prior to the formal survey, an exploratory qualitative inquiry was conducted. Through a review of relevant literature and semi-structured interviews with 15 urban employees engaged in high-pressure occupations, key environmental attributes in rural tourism settings associated with restorative experiences were initially identified. This process ensured the content validity of the subsequent quantitative measurement scales within the specific rural context and refined a broad set of environmental factors into core dimensions emphasized by Attention Restoration Theory.

Subsequently, structural equation modeling (SEM) was employed to test the overall theoretical model and research hypotheses. As a multivariate statistical technique capable of simultaneously estimating measurement and structural models, SEM allows for the systematic examination of complex relationships among multiple independent variables, mediators, and dependent variables while controlling for measurement error (34). Although SEM effectively evaluates linear structural paths among constructs, the relationship between restorative experiences and subsequent behavioral intentions in rural tourism contexts may involve nonlinear or asymmetric patterns. To address this limitation, ANN analysis was introduced. ANN does not require assumptions of normality or linearity and is capable of capturing more complex data structures (35). Furthermore, sensitivity analysis within the ANN framework enables the identification of the relative importance of predictor variables, thereby offering more precise prioritization guidance regarding job crafting intention. The specific analytical procedures are described as follows:

- Missing data treatment and software selection. Prior to formal analysis, the dataset was screened and cleaned. Because the questionnaire required mandatory responses and responses with excessively short completion times were excluded, the proportion of missing data was below 1%. Mean substitution was applied to address missing values. Statistical analyses were conducted using SPSS 26.0 and AMOS 24.0, while ANN modeling was implemented *via* the Neural Networks module in SPSS.
- SEM estimation and model testing. Parameters were estimated using the Maximum Likelihood (ML) method. To examine the significance of mediation effects, a bootstrap procedure with 5,000 resamples was performed, and 95% confidence intervals were calculated. Moderation effects were tested using interaction product terms to construct the moderated structural model.
- ANN architecture and overfitting control. A multilayer perceptron (MLP) network was employed and trained using the backpropagation algorithm. The network consisted of an input layer, a hidden layer, and an output layer. The hidden layer utilized a hyperbolic tangent activation function, while the output layer employed an identity activation function. To prevent overfitting, ten-fold cross-validation was implemented. The dataset was partitioned into 90% training and 10% testing subsets, and model generalizability was evaluated by comparing the root mean square error (RMSE) between training and testing samples.

### 3.2 Questionnaire design and variable measurement

This study collected empirical data through a structured questionnaire developed in accordance with rigorous scale development and translation procedures. To ensure measurement validity, the core constructs were adapted from well-established scales previously published in both domestic and international research, with contextual modifications tailored to the rural tourism setting. Prior to the formal survey, a pilot study was conducted in August 2025, yielding 139 valid responses. Reliability analysis and exploratory factor analysis were performed, and items with low factor loadings or ambiguous wording were removed. The finalized questionnaire comprised eight dimensions with a total of 32 items (see Table 1 for detailed measurements).

**Table 1:** Questionnaire variables and item design.

Variable dimension	Item	Source of the original scale
*Distance from stressors* *(DFS)*	Take a break from work pressures	Kaplan et al. (36)
	Escape life's troubles	Osbaldiston (37)
	Step away from your usual routine	Korpela et al. (38)
	Minimize work distractions	Rita Berto (39)
*High visual authenticity* *(HVA)*	Visual Authenticity	Li et al. (40)
	Rural landscapes presented in their natural state without artificial embellishment	Shen et al. (41)
	Immersive Visual Experience	Wen et al. (42)
	Free from staged or artificial presentation	Kardan et al. (43)
*Experience continuity* *(EC)*	Seamless holistic experience	Gupta et al. (44); AC Bender. et al. (45)
	Continuously exploring possibilities	
	Natural spatial transitions	
	Never monotonous over time	
*Personal compatibility* *(PC)*	Satisfy relaxation needs	Van Den Berg et al. (46); Chen et al. (47)
	Freely match activities	
	Meet psychological expectations	
	Highly compatible with the environment	
*Psychological restoration* *(PSY)*	Reduced psychological stress	Qiu et al. (48); Neale et al. (49)
	Emotions tend toward calmness	
	Pleasant and relaxing experience	
	Restoration of mental tranquility	
*Physiological restoration* *(PHY)*	Relief from physical fatigue	Berto (39); Li et al. (50)
	Sense of bodily relaxation	
	Restoration of physical strength and energy	
	Improvement in physical condition	
*Tourist behavior patterns* *(TBP)*	Low-intensity static activities	Chen et al. (47)
	Slow-paced, lingering behavior	
	High-intensity physical activities	
	Alternating dynamic and static patterns	
*Job crafting intention* *(JCI)*	Proactively adjust work approaches	Wang et al. (51)
	Experiment with new work methods	
	Reshape work rhythms	
	Enhance work experiences	

All measurement items were assessed using a five-point Likert scale, ranging from 1 (“strongly disagree”) to 5 (“strongly agree”), with higher scores indicating stronger perceived intensity or intention. In the questionnaire instructions, respondents were explicitly asked to base their evaluations on their most recent rural tourism experience within the past 6 months. This approach was adopted to minimize recall bias and to ensure that responses were grounded in a specific and concrete tourism episode rather than a generalized impression.

### 3.3 Questionnaire distribution and data collection

**Figure 3:**
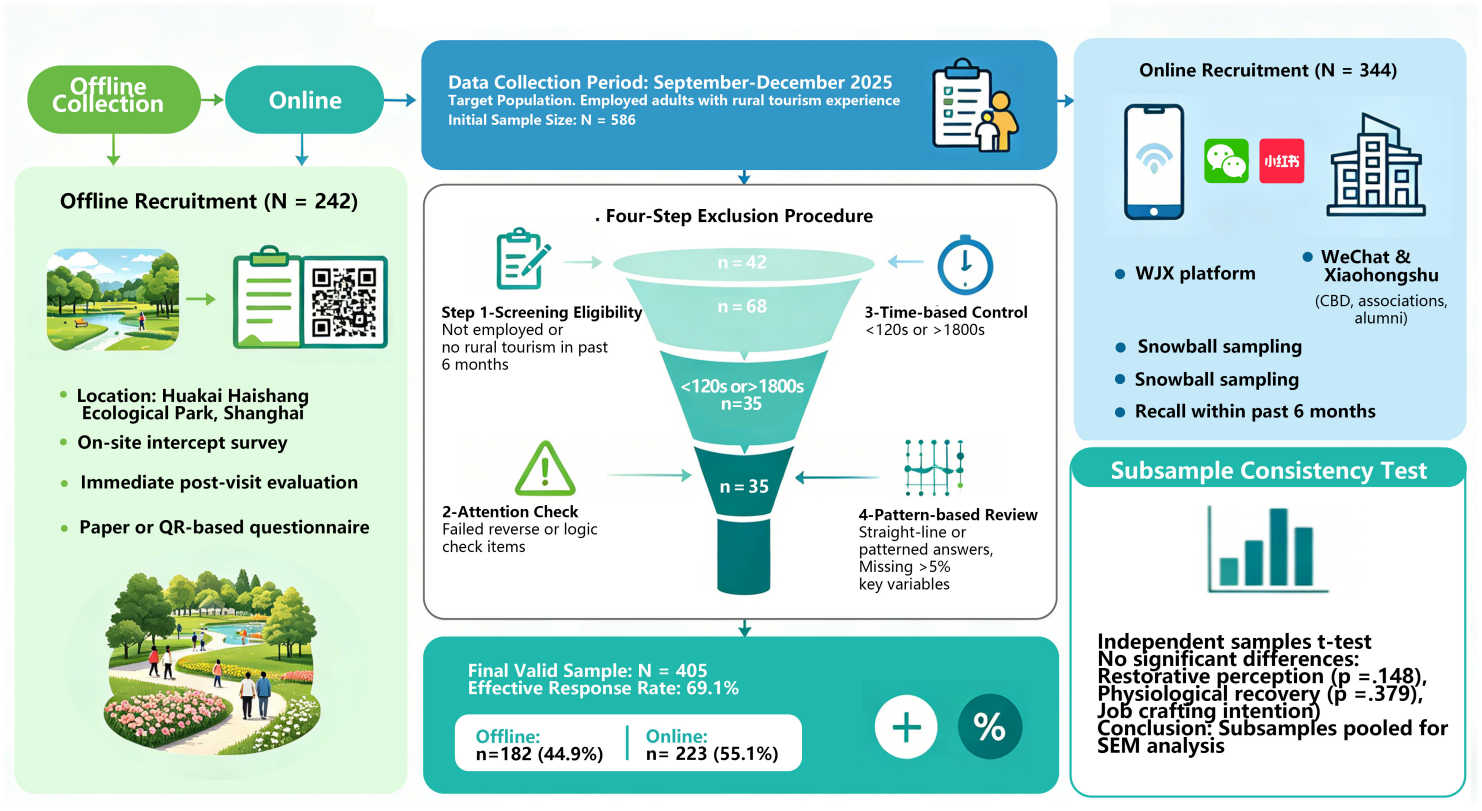
Questionnaire distribution and screening procedure.

This study was conducted in strict accordance with the ethical principles outlined in the Declaration of Helsinki and received formal approval from the Human Research Ethics Committee of Donghua University (Approval No. RLSSZYJ202509280057). Prior to the commencement of data collection, all participants were fully informed of the study's objectives, anonymity safeguards, and intended use of data, and written informed consent was obtained from each respondent. Data were collected between September and December 2025. The target population was strictly limited to employed individuals with prior rural tourism experience. To ensure the scientific rigor of the findings and the integrity of the sample, a systematic questionnaire distribution and screening procedure was implemented (Figure 3).

#### 3.3.1 Questionnaire distribution and initial recruitment

Data collection was conducted through a combination of on-site field surveys and targeted online recruitment, yielding a total of 586 initial responses.

**Figure 4:**
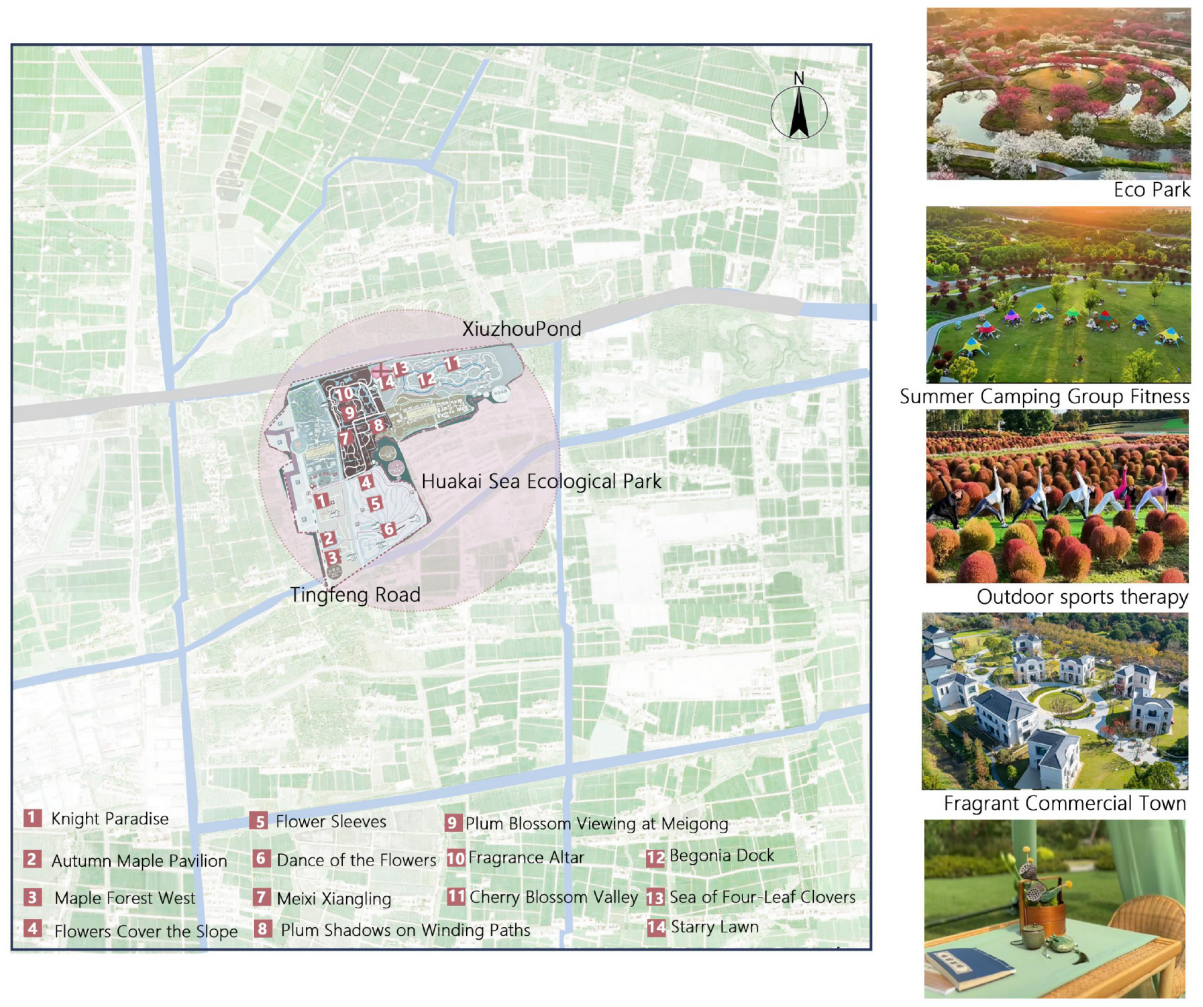
Locations for distributing paper surveys. Images (Eco Park, Summer Camping Group Fitness, Outdoor sports therapy, Fragrant Commercial Town) by Huakaihai Ecological Park (http://xhslink.com/o/7eyUngXLOhg) reproduced with permission.

- Offline channel (*N* = 242): surveyors approached visitors in the leisure area of Huakai Haishang Ecological Park in Jinshan District, Shanghai, using an intercept method and inviting them to complete either a QR-code-based or paper questionnaire. Respondents in this group evaluated their experiences based on immediate rural tourism exposure. Located in Xiujing Village, Zhujing Town, Jinshan District, Huakai Haishang Ecological Park lies in the southwestern urban–rural fringe of Shanghai and represents one of the district's key rural leisure tourism destinations. It has been designated as a National 4A Tourist Attraction and functions as a flower-themed ecological park integrating floral appreciation, ecological agriculture, science education, and leisure vacation. Representative landscape nodes within the park are shown in Figure 4. Its integrated environmental characteristics—combining natural scenery, multisensory floral landscapes, and local rural ambiance—closely align with the research focus on perceived restorative environments, physiological and psychological recovery, and job crafting intention. Accordingly, it was selected as the primary offline site for questionnaire administration.
- Online channel (*N* = 344): to broadly reach urban professionals working under high-pressure conditions, an electronic questionnaire was developed using the Wenjuanxing (WJX) platform and disseminated through social media channels (WeChat, Xiaohongshu) as well as targeted professional communities (e.g., industry associations, CBD workplace groups, and alumni networks). A combination of purposive sampling and snowball sampling was employed, leveraging the relative closure of occupational communities to ensure respondents' employment status. To reduce recall bias inherent in retrospective surveys, participants were explicitly instructed to base their responses on the most memorable and representative rural tourism experience within the past 6 months.

#### 3.3.2 Sample screening and exclusion criteria

To obtain the final sample of 405 valid responses from the initial dataset, a rigorous four-step exclusion procedure was implemented:

- Eligibility screening: two mandatory filter questions were placed at the beginning of the questionnaire: “Are you currently employed?” and “Have you participated in at least one rural tourism activity within the past 6 months?” A total of 42 ineligible respondents were automatically terminated and excluded.
- Attention check: reverse-coded items and logical verification questions (e.g., “Please select ‘strongly agree' for this item”) were embedded in the questionnaire. A total of 68 responses that failed the attention checks were removed.
- Time-based exclusion: based on pilot testing, completing the questionnaire required at least 180 s. Responses completed in an unusually short time (e.g., less than 120 s) or an excessively long time (e.g., more than 1,800 s) were deemed invalid, resulting in the exclusion of 35 cases.
- Pattern-based exclusion: questionnaires exhibiting highly regular response patterns (e.g., straight-lining or uniform scaling across items) or containing more than 5% missing values in key variables were removed, totaling 36 cases.

#### 3.3.3 Final sample composition

Following the aforementioned screening procedures, a total of 405 valid questionnaires were retained, yielding an overall effective response rate of 69.1%. According to Bentler and Chou (52), the recommended ratio of sample size to measurement items in structural equation modeling (SEM) ranges from 5:1 to 10:1. Given that the present model includes 32 measurement items, the theoretically required sample size would range between 160 and 320 cases. The final sample of 405 valid responses substantially exceeds this benchmark. Among the 405 cases, 182 were collected offline (44.9%) and 223 online (55.1%). To assess potential systematic differences between data collection channels, independent samples *t*-tests were conducted comparing the two subsamples. The results indicated no significant differences between online and offline respondents in perceived restorative environment (*t* = 1.45, *p* = 0.148 > 0.05), physiological recovery (*t* = 0.88, *p* = 0.379 > 0.05), or job crafting intention (*t* = −1.12, *p* = 0.263 > 0.05). These findings suggest that the data collection mode did not introduce significant bias into the structural model, and that the overall sample demonstrates satisfactory homogeneity. Accordingly, the two datasets were combined for subsequent analyses.

## 4 Structural equation modeling analysis

### 4.1 Reliability and validity testing

To ensure the reliability and validity of the research data, the measurement model was evaluated in terms of internal consistency and convergent validity, with results presented in Table 2. Regarding reliability, Cronbach's α coefficients for all latent constructs ranged from 0.811 to 0.917, while composite reliability (CR) values ranged from 0.814 to 0.918. Both indicators exceed the threshold of 0.70 recommended by Nunnally (53), indicating strong internal consistency of the scales. In terms of convergent validity, standardized factor loadings for all measurement items ranged from 0.629 to 0.875, exceeding the 0.50 criterion suggested by Murtagh et al. (54) and reaching statistical significance. This demonstrates that the items adequately represent their respective latent constructs. Furthermore, the average variance extracted (AVE) values for all constructs ranged from 0.524 to 0.737, surpassing the 0.50 benchmark proposed by Fornell and Larcker (55), thereby supporting satisfactory convergent validity of the measurement model.

**Table 2:** Reliability and convergent validity of each variable.

Construct		Estimate	Cronbach's α	AVE	CR
*Distance from stressors* *(DFS)*	DFS1	0.786	0.877	0.642	0.878
	DFS2	0.773			
	DFS3	0.863			
	DFS4	0.780			
*High visual authenticity* *(HVA)*	HVA1	0.793	0.855	0.596	0.855
	HVA2	0.768			
	HVA3	0.759			
	HVA4	0.768			
*Experience continuity* *(EC)*	EC1	0.679	0.811	0.523	0.814
	EC2	0.777			
	EC3	0.701			
	EC4	0.732			
*Personal compatibility* *(PC)*	PC1	0.802	0.826	0.542	0.824
	PC2	0.799			
	PC3	0.626			
	PC4	0.702			
*Psychological restoration* *(PSY)*	PSY1	0.771	0.865	0.620	0.867
	PSY2	0.860			
	PSY3	0.795			
	PSY4	0.718			
*Physiological restoration* *(PHY)*	PHY1	0.865	0.895	0.688	0.898
	PHY2	0.796			
	PHY3	0.825			
	PHY4	0.830			
*Tourist behavior patterns* *(TBP)*	TBP1	0.761	0.833	0.555	0.833
	TBP2	0.753			
	TBP3	0.736			
	TBP4	0.728			
*Job crafting intention* *(JCI)*	JCI1	0.850	0.917	0.737	0.918
	JCI2	0.838			
	JCI3	0.875			
	JCI4	0.871			

As shown in Table 3, the Kaiser–Meyer–Olkin (KMO) measure for this study reached 0.935, substantially exceeding the recommended threshold of 0.80. According to Kaiser's classification criteria (56), this value indicates a “marvelous” level of sampling adequacy, suggesting that the sample size and inter-item correlations are sufficient to support factor analysis. Bartlett's test of sphericity yielded a chi-square value of 7,959.275 with 496 degrees of freedom, and the result was statistically significant (*p* < 0.001). This finding indicates that the correlation matrix significantly deviates from an identity matrix and that strong correlations exist among the measurement items, thereby rejecting the null hypothesis of sphericity. Collectively, these results demonstrate that the data are well suited for subsequent exploratory and confirmatory factor analyses, providing a solid statistical foundation for constructing the latent structural model.

**Table 3:** KMO and Bartlett's sphericity test results.

KMO		0.935
Bartlett's sphericity	spherical test	7,959.275
	*df*-value	496
	*p*-value	0.000

### 4.2 Fornell-Larcker criterion test

As presented in Table 4, the square roots of the average variance extracted (AVE) for all latent constructs (diagonal elements) exceed their corresponding inter-construct correlations (off-diagonal elements). For instance, the square root of AVE for job crafting intention (JCI) is 0.774, which is greater than its correlations with psychological recovery, physiological recovery, distance from stressors, and other constructs (ranging from 0.231 to 0.571). Similarly, the constructs of physiological recovery (0.868), psychological recovery (0.821), distance from stressors (0.979), high visual authenticity (0.861), personal compatibility (0.684), experience continuity (0.777), and tourist behavior patterns (0.816) all satisfy the same criterion. Overall, according to the Fornell–Larcker criterion (55), the latent constructs in this study demonstrate adequate discriminant validity, indicating that the measurement model possesses a sound foundation in terms of construct distinctiveness.

**Table 4:** Fornell-Larcker test.

Variable dimension	JCI	PHY	PSY	DFS	HVA	PC	EC	TBP
*JCI*	0.774							
*PHY*	0.549	0.868						
*PSY*	0.552	0.476	0.821					
*DFS*	0.571	0.498	0.525	0.979				
*HVA*	0.511	0.470	0.461	0.464	0.861			
*PC*	0.498	0.437	0.454	0.473	0.428	0.684		
*EC*	0.551	0.479	0.499	0.545	0.469	0.481	0.777	
*TBP*	0.231	0.166	0.257	0.202	0.174	0.250	0.364	0.816

The diagonal elements represent the square root of the AVE value for each variable. 2. The off-diagonal elements represent the correlations between constructs. 3. DFS, distance from stressors; HVA, high visual authenticity; EC, experience continuity; PC, personal compatibility; PSY, psychological restoration; PHY, physiological restoration; TBP, tourist behavior patterns; JCI, job crafting intention.

### 4.3 Heterotrait-Monotrait ratio test

As shown in Table 5, the heterotrait–monotrait ratio (HTMT) values among all pairs of latent constructs range from 0.198 to 0.710. The highest value, observed between job crafting intention and experience continuity, is 0.710, which is well below the commonly accepted discriminant validity thresholds of 0.85 (and 0.90). According to the HTMT criterion proposed by Henseler et al. (57), no excessively high heterotrait–monotrait ratios are present among the constructs. This indicates that the latent variables in this study are empirically distinct from one another, providing further evidence of satisfactory discriminant validity in the measurement model.

**Table 5:** Discriminant validity test results (HTMT ratio).

Variable dimension	JCI	PHY	PSY	DFS	HVA	PC	EC	TBP
*JCI*	1.000							
*PHY*	0.670	1.000						
*PSY*	0.692	0.563	1.000					
*DFS*	0.656	0.541	0.586	1.000				
*HVA*	0.626	0.543	0.549	0.506	1.000			
*PC*	0.684	0.567	0.606	0.578	0.558	1.000		
*EC*	0.710	0.584	0.625	0.625	0.574	0.661	1.000	
*TBP*	0.290	0.198	0.314	0.226	0.207	0.334	0.457	1.000

DFS, distance from stressors; HVA, high visual authenticity; EC, experience continuity; PC, personal compatibility; PSY, psychological restoration; PHY, physiological restoration; TBP, tourist behavior patterns; JCI, job crafting intention.

### 4.4 Structural model evaluation

As reported in Table 6, the confirmatory factor analysis (CFA) model demonstrates an overall satisfactory fit. The chi-square statistic is 785.492 with 436 degrees of freedom, yielding a χ^2^/df (CMIN/DF) ratio of 1.802, which is below the recommended threshold of 3.0 and indicates acceptable model parsimony. Regarding absolute fit indices, the goodness-of-fit index (GFI = 0.896) and adjusted goodness-of-fit index (AGFI = 0.875) both exceed the 0.80 benchmark. The root mean square error of approximation (RMSEA = 0.045) is well below the 0.08 threshold proposed by Browne and Cudeck (58), indicating a strong model fit. In terms of incremental fit indices, the incremental fit index (IFI = 0.955), normed fit index (NFI = 0.904), Tucker–Lewis index (TLI = 0.948), and comparative fit index (CFI = 0.955) all surpass the recommended 0.90 criterion suggested by Hu and Bentler (59). Taken together, these indices suggest that the measurement model exhibits a sound structural configuration and satisfactory fit, providing a reliable basis for subsequent structural equation modeling analyses.

**Table 6:** Model fit for confirmatory factor analysis.

Fit index	Acceptable range	Measurement value
*CMIN*		785.492
*DF*		436
*CMIN/DF*	< 3	1.802
*GFI*	> 0.8	0.896
*AGFI*	> 0.8	0.875
*RMSEA*	< 0.08	0.045
*IFI*	> 0.9	0.955
*NFI*	> 0.9	0.904
*TLI(NNFI)*	> 0.9	0.948
*CFI*	> 0.9	0.955

### 4.5 Hypothesis testing

**Figure 5:**
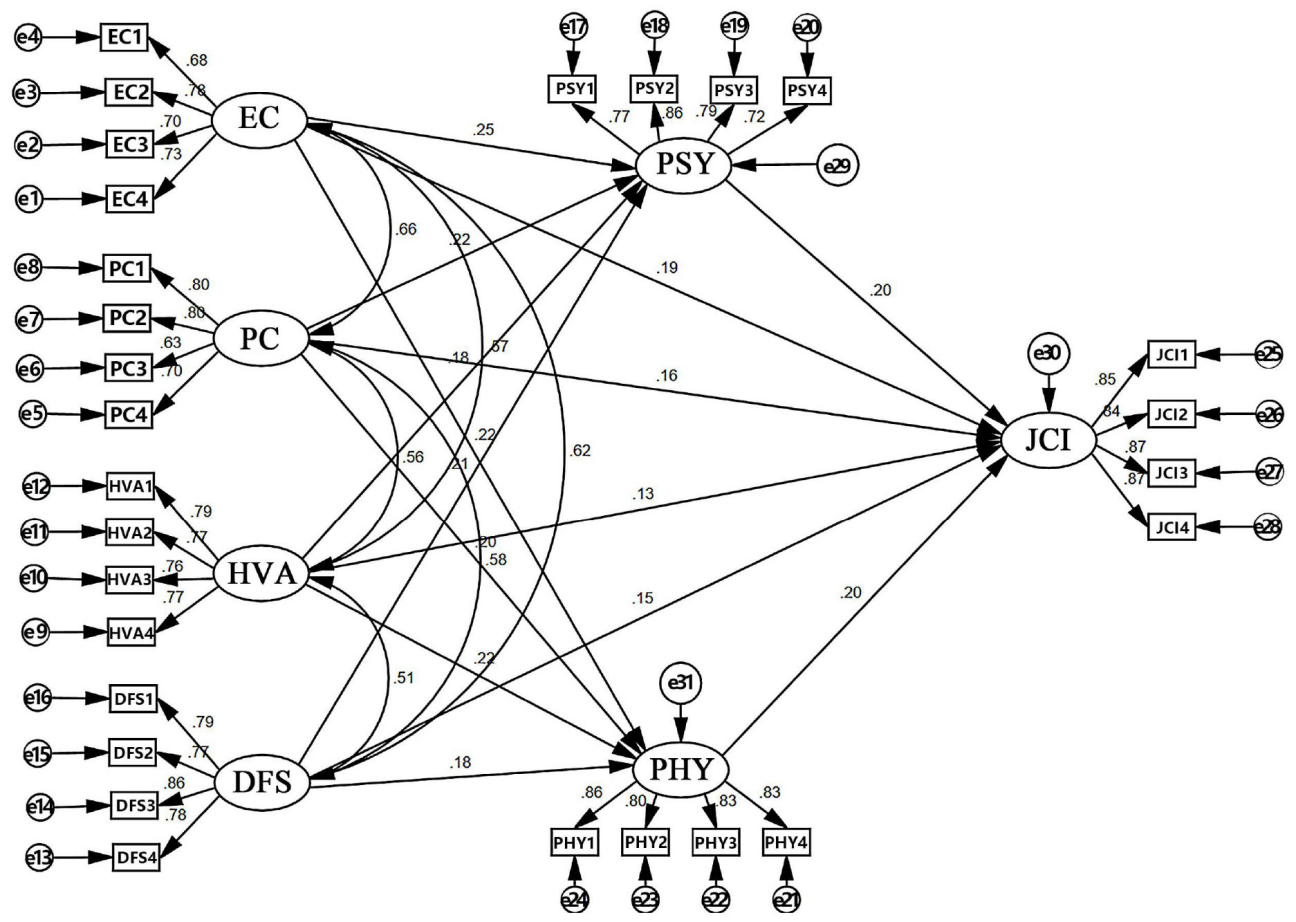
Structural equation modeling diagram.

As shown in Table 7 and Figure 5, all hypothesized paths proposed in this study are supported by the empirical data. First, distance from stressors, visual authenticity, experiential continuity, and personal compatibility each exert a significant positive effect on job crafting intention (H1–H4, β = 0.122–0.193, *p* < 0.05), indicating that perceived restorative environments directly enhance individuals' intention to proactively optimize their work. Second, both psychological recovery and physiological recovery have significant positive effects on job crafting intention (H5, H6; β = 0.195 and 0.188, respectively; *p* < 0.001). Moreover, the path coefficients from the four environmental perception dimensions to both psychological and physiological recovery are all positive and statistically significant (H5a–H5d, H6a–H6d; *p* < 0.01). These findings suggest that restorative environments in rural tourism strengthen individuals' job crafting intention by simultaneously promoting psychological and physiological recovery. Overall, the results validate the parallel mediation mechanism of “perceived restorative environment → psychological/physiological recovery → job crafting intention” and provide empirical support for the proposed S-O-R theoretical framework.

**Table 7:** Hypothesis testing analysis.

Hypothesis				Estimate	S.E.	C.R.	*P*	Testing the hypothesis
*H1*	JCI	< –	DFS	0.134	0.047	2.875	0.004^**^	Established
*H2*	JCI	< –	HVA	0.122	0.048	2.550	0.011^*^	Established
*H3*	JCI	< –	EC	0.193	0.066	2.935	0.003^**^	Established
*H4*	JCI	< –	PC	0.174	0.064	2.725	0.006^**^	Established
*H5*	JCI	< –	PSY	0.195	0.054	3.637	0.000^***^	Established
*H5a*	PSY	< –	DFS	0.170	0.061	2.775	0.006^**^	Established
*H5b*	PSY	< –	HVA	0.218	0.063	3.435	0.000^***^	Established
*H5c*	PSY	< –	EC	0.235	0.085	2.746	0.006^**^	Established
*H5d*	PSY	< –	PC	0.229	0.083	2.744	0.006^**^	Established
*H6*	JCI	< –	PHY	0.188	0.048	3.943	0.000^***^	Established
*H6a*	PHY	< –	DFS	0.197	0.059	3.341	0.000^***^	Established
*H6b*	PHY	< –	HVA	0.173	0.061	2.853	0.004^**^	Established
*H6c*	PHY	< –	EC	0.256	0.082	3.125	0.002^**^	Established
*H6d*	PHY	< –	PC	0.247	0.080	3.076	0.002^**^	Established

^***^ indicates *p* < 0.001;

^**^ indicates *p* < 0.01;

^*^ indicates *p* < 0.05; DFS, distance from stressors; HVA, high visual authenticity; EC, experience continuity; PC, personal compatibility; PSY, psychological restoration; PHY, physiological restoration; TBP, tourist behavior patterns; JCI, job crafting intention.

### 4.6 Parallel mediation analysis of psychological and physiological recovery

As shown in Table 8, the four dimensions of perceived restorative environment (DFS, HVA, EC, and PC) exert significant parallel mediating effects on job crafting intention (JCI) through psychological recovery (PSY) and physiological recovery (PHY). Specifically, the lower and upper bounds of all indirect effect confidence intervals are positive (e.g., DFS → PSY → JCI: 0.007–0.094; DFS → PHY → JCI: 0.004–0.078; EC → PSY → JCI: 0.009–0.122), with all *p*-values below 0.05. These results indicate that both psychological and physiological recovery function as significant mediators across the proposed pathways. Meanwhile, the four direct paths (DFS → JCI, HVA → JCI, EC → JCI, and PC → JCI) remain statistically significant, and all total effects are positive and significant. Among them, experiential continuity (EC) exhibits the largest total effect, followed by personal compatibility (PC), distance from stressors (DFS), and high visual authenticity (HVA). This pattern suggests that psychological and physiological recovery partially mediate the relationship between perceived restorative environment and job crafting intention. Furthermore, the indirect effects transmitted through psychological recovery are generally slightly stronger than those through physiological recovery, indicating that psychological restoration plays a more prominent role in enhancing individuals' job crafting intention.

**Table 8:** Parallel mediating effects analysis of psychological recovery and physiological recovery.

Mediation Path	Effect	Estimate	Lower	Upper	*P*
*DFS—PSY—JCI*	Indirect Effect	0.043	0.007	0.094	0.015
*DFS—PHY—JCI*	Indirect Effect	0.036	0.004	0.078	0.027
*DFS—JCI*	Direct Effect	0.151	0.021	0.284	0.022
*DFS—JCI*	Total Effect	0.230	0.097	0.357	0.003
*HVA—PSY—JCI*	Indirect Effect	0.036	0.001	0.087	0.037
*HVA—PHY—JCI*	Indirect Effect	0.043	0.005	0.091	0.017
*HVA—JCI*	Direct Effect	0.129	0.010	0.240	0.036
*HVA—JCI*	Total Effect	0.208	0.068	0.336	0.004
*EC—PSY—JCI*	Indirect Effect	0.050	0.009	0.122	0.008
*EC—PHY—JCI*	Indirect Effect	0.044	0.005	0.094	0.022
*EC—JCI*	Direct Effect	0.193	0.025	0.364	0.025
*EC—JCI*	Total Effect	0.287	0.124	0.469	0.001
*PC—PSY—JCI*	Indirect Effect	0.045	0.003	0.106	0.032
*PC—PHY—JCI*	Indirect Effect	0.040	0.003	0.088	0.034
*PC—JCI*	Direct Effect	0.163	0.024	0.296	0.022
*PC—JCI*	Total Effect	0.249	0.099	0.388	0.002

DFS, distance from stressors; HVA: high visual authenticity; EC, experience continuity; PC, personal compatibility; PSY, psychological restoration; PHY, physiological restoration; TBP, tourist behavior patterns; JCI, job crafting intention.

### 4.7 Moderating effect analysis of tourist behavioral patterns

**Figure 6:**
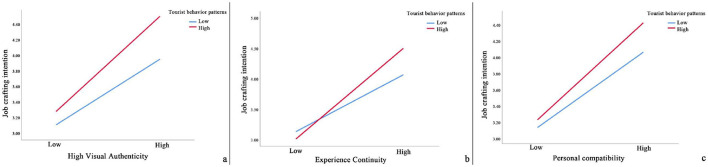
The moderating role of visitor behavior patterns in the relationship between restorative environmental perception and job crafting intention **(a)** Moderating role of visitor behavior patterns in the relationship between high visual authenticity and Job crafting intention; **(b)** Moderating role of visitor behavior patterns in the relationship between experiential continuity and Job crafting intention; **(c)** Moderating role of visitor behavior patterns in the relationship between Personal compatibility and Job crafting intention.

To examine the moderating role of tourist behavior patterns in the relationships between the independent variables and job crafting intention, interaction terms were incorporated into the structural equation model and moderation analyses were conducted. The results are presented in Table 9 and Figure 6. The findings indicate that the interaction between visual authenticity and tourist behavior patterns exerts a significant positive moderating effect on job crafting intention [Effect Size = 0.097, *T* = 2.607, *p* = 0.009, 95% CI (0.024, 0.170)], supporting Hypothesis H7b. The interaction between experience continuity and tourist behavior patterns shows the strongest moderating effect [Effect Size = 0.146, *T* = 4.183, *p* < 0.001, 95% CI (0.077, 0.215)], supporting Hypothesis H7c. Similarly, the interaction between personal compatibility and tourist behavior patterns demonstrates a significant positive moderating effect [Effect Size = 0.075, *T* = 2.018, *p* = 0.044, 95% CI (0.002, 0.149)], providing support for Hypothesis H7d. These results suggest that higher levels of tourist behavioral engagement amplify the positive associations between authenticity, compatibility, experience continuity, and job crafting intention.

**Table 9:** Analysis of the moderating effects of visitor behavior patterns.

Hypothesis	Effect type	Effect size	Standard deviation	T	*p*	Confidence interval	
						LLCI	ULCI
*H7b*	HVA × TBP → JCI	0.097	0.037	2.607	0.009	0.024	0.170
*H7c*	EC × TBP → JCI	0.146	0.035	4.183	0.000	0.077	0.215
*H7d*	PC × TBP → JCI	0.075	0.037	2.018	0.044	0.002	0.149

HVA, high visual authenticity; EC, experience continuity; PC, personal compatibility; TBP, tourist behavior patterns; JCI, job crafting intention.

In contrast, the interaction between distance from stressors and tourist behavior patterns (H7a) does not reach statistical significance (*p* > 0.05, with confidence intervals crossing zero), indicating that tourist behavior patterns do not significantly moderate the relationship between distance from stressors and job crafting intention. Overall, tourist behavior patterns exhibit partial moderating effects across three of the four core dimensions, excluding distance from stressors.

## 5 ANN neural network analysis

**Figure 7:**
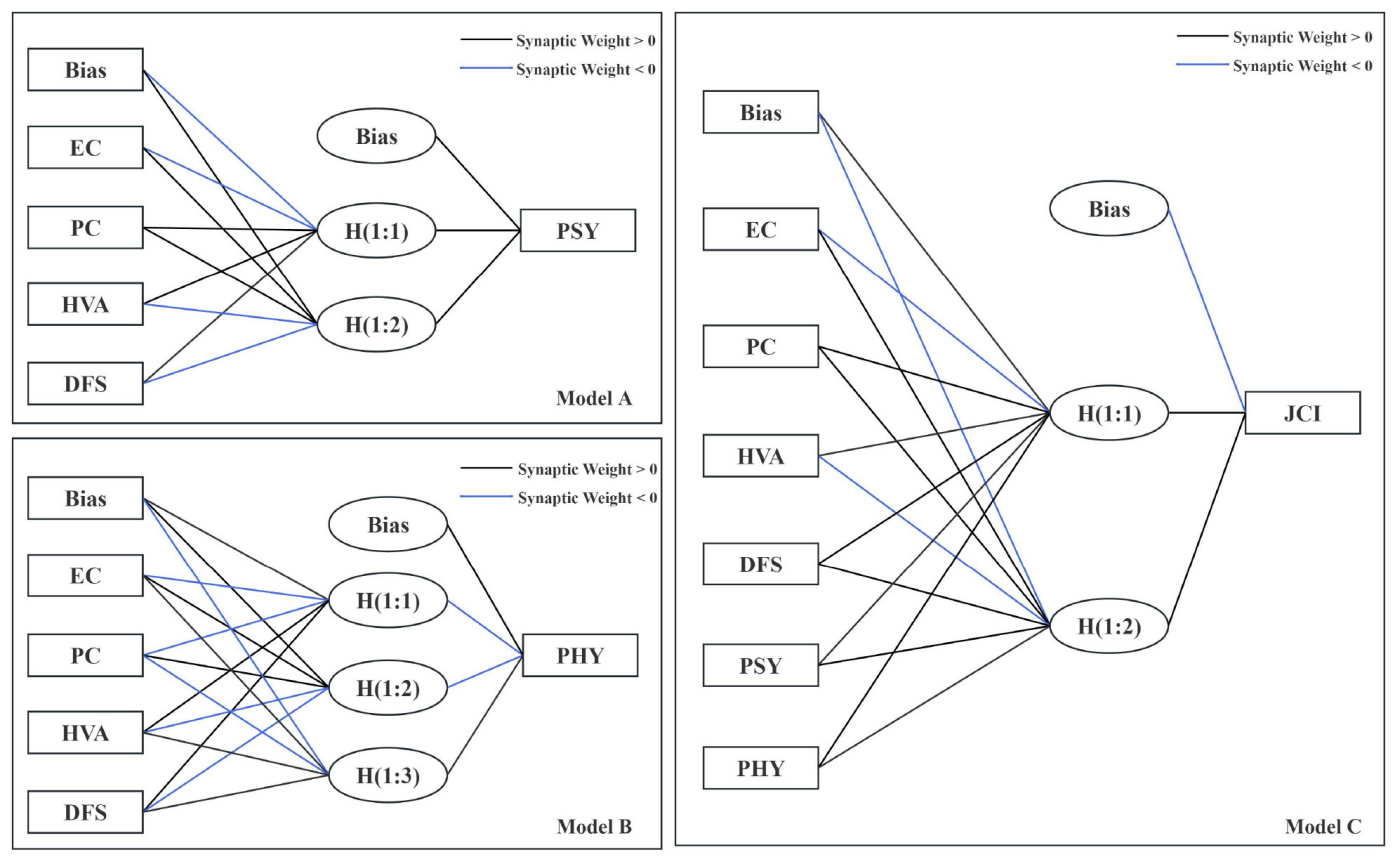
Artificial neural network model construction.

Based on the significant paths validated by SEM, this study further constructs a Multilayer Perceptron (MLP) artificial neural network model to complement the structural equation modeling analysis from a prediction-oriented nonlinear perspective (see Figure 7). According to the significant independent variables identified in the SEM results as input nodes, three sub-models are developed. Models A and B share the same input layer, including Experience Continuity (EC), Personal Congruence (PC), High Visual Authenticity (HVA), and Degree of Being Away from Stressors (DFS), to predict Psychological Restoration (PSY) and Physiological Restoration (PHY), respectively. Model C further incorporates environmental and restoration variables (EC, PC, HVA, DFS, PSY, and PHY) into the input layer to comprehensively predict Job Crafting Intention (JCI).

Each sub-model contains one hidden layer to capture potential nonlinear relationships. The number of neurons in the hidden layer is automatically optimized by the system, and the Sigmoid activation function is employed to enhance nonlinear mapping capability. The output layer uses the Identity activation function, which is suitable for predicting continuous dependent variables. Model training is conducted using the Backpropagation Algorithm to optimize weights. To enhance model stability and prevent overfitting, a 10-fold cross-validation method is applied for repeated training and validation. Model performance is evaluated using Mean Squared Error (MSE) and relative importance indices. Through these parameter settings and cross-validation procedures, the ANN analysis ensures strong predictive stability and replicability.

### 5.1 Root mean square error validation

As shown in Table 10, the root mean square error (RMSE) values for the three artificial neural network models range overall between 0.20 and 0.30 across both the training and testing datasets, indicating relatively low prediction errors. For Model A, which uses psychological recovery (PSY) as the output variable, the average RMSE values for the training and testing sets are 0.288 and 0.260, respectively. This suggests that the four input variables—distance from stressors (DFS), high visual authenticity (HVA), personal compatibility (PC), and experiential continuity (EC)—demonstrate satisfactory fitting and generalization capability in predicting psychological recovery. Model B, with physiological recovery (PHY) as the output, yields average RMSE values of 0.277 (training) and 0.254 (testing), performing comparably to Model A. This indicates that the same environmental perception indicators also exhibit stable predictive power for physiological recovery. In comparison, Model C—whose input layer further incorporates PSY and PHY and whose output is job crafting intention (JCI)—achieves the lowest average RMSE in the testing set (0.202), outperforming Models A and B. This finding suggests that incorporating recovery-related outcome variables substantially improves predictive accuracy for job crafting intention. Although some fluctuations in RMSE are observed across individual networks (ANN1–ANN10), the overall average results indicate that the integrated SEM–ANN approach demonstrates strong predictive performance and acceptable robustness.

**Table 10:** Root mean square error test.

Variable dimension	Model A		Model B		Model C	
	Input: DFS, HVA, PC, EC		Input: DFS, HVA, PC, EC		Input: DFS, HVA, PC, EC, PSY, PHY	
	Output: PSY		Output: PHY		Output: JCI	
*Neural network*	Training	Testing	Training	Testing	Training	Testing
5 *ANN1*	0.266	0.309	0.267	0.221	0.196	0.188
*ANN2*	0.290	0.222	0.298	0.206	0.175	0.147
*ANN3*	0.297	0.385	0.253	0.205	0.195	0.130
*ANN4*	0.272	0.240	0.292	0.289	0.187	0.116
*ANN5*	0.276	0.366	0.261	0.302	0.188	0.222
*ANN6*	0.286	0.232	0.275	0.319	0.194	0.217
*ANN7*	0.315	0.336	0.278	0.169	0.243	0.106
*ANN8*	0.267	0.152	0.261	0.278	0.211	0.204
*ANN9*	0.270	0.256	0.297	0.276	0.247	0.238
*ANN10*	0.319	0.148	0.283	0.278	0.118	0.114
*Mean*	0.286	0.265	0.277	0.254	0.202	0.168
*SD*	0.139	0.288	0.126	0.223	0.156	0.225

JCI, job crafting intention; PHY, physiological restoration; PSY, psychological restoration; DFS, distance from stressors; HVA, high visual authenticity; PC, personal compatibility; EC, experience continuity.

To further clarify the advantages of artificial neural networks (ANN) in capturing complex nonlinear relationships, this study compares the predictive performance of the traditional structural equation modeling (SEM) approach with that of ANN models (Table 11). In terms of predictive accuracy, the comparison results indicate that the average RMSE values of the ANN test sets across the three sub-models (0.202–0.260) are all lower than the corresponding error indices of the SEM models. Notably, in Model C, which predicts the core dependent variable “Job Crafting Intention (JCI),” the ANN demonstrates exceptionally high fitting accuracy (RMSE = 0.202). This finding suggests that ANN, through its self-learning capability, can capture complex patterns in the data that may be overlooked under the linear assumptions of SEM. Regarding explanatory power and robustness, although SEM confirms the hypothesized logical directions through path analysis (Table 7), its interpretation of interactions among variables is often constrained by linear correlations. In contrast, after incorporating mediating variables (psychological and physiological restoration), the ANN model achieves a further reduction in prediction error (Model C) compared with models that include only environmental perception variables (Models A and B).

**Table 11:** Comparison of predictive performance between SEM and ANN.

*Number*	Output variable	SEM coefficient of determination (R^2^)	SEM (RMSE)	ANN (RMSE)	Explanation of the improved predictive performance of ANN
*Model A*	PSY	0.518	0.694	0.260	The ANN error is significantly lower than that of the SEM
*Model B*	PHY	0.463	0.733	0.254	The ANN model is more robust
*Model C*	JCI	0.700	0.548	0.202	It achieves the highest prediction accuracy and exhibits distinct nonlinear characteristics

JCI: Job crafting intention; PHY, physiological restoration; PSY, psychological restoration. 2. The RMSE for the SEM model was calculated as the square root of (1–R^2^), based on the standardized results.

In summary, the introduction of ANN serves not merely as a cross-validation of SEM's linear results; its core contributions are twofold. First, it demonstrates that the influence of restorative environments on job crafting is not a simple linear aggregation but rather a complex mapping characterized by nonlinear features. Second, it provides more precise predictive capability than SEM, thereby offering more reliable decision-support insights for rural tourism destinations seeking to optimize visitor experiences and promote behavioral transformation.

### 5.2 Sensitivity analysis

As shown in Table 12, in Model A—where psychological recovery (PSY) is the output variable—experiential continuity (EC) exhibits the highest normalized importance (100.000%), making it the most influential predictor of psychological recovery. It is followed by personal compatibility (PC, 80.263%), distance from stressors (DFS, 76.316%), and high visual authenticity (HVA, 72.368%). The latter three show relatively similar levels of importance, each demonstrating substantial predictive power for psychological recovery. In Model B, with physiological recovery (PHY) as the output, EC remains the most important predictor (100.000%), followed by HVA (71.772%), PC (67.868%), and DFS (60.961%). These results indicate that experiential continuity plays a central role in both psychological and physiological recovery processes.

**Table 12:** Analysis of the importance of normalization in artificial neural network models.

Neural network		ANN1	ANN2	ANN3	ANN4	ANN5	ANN6	ANN7	ANN8	ANN9	ANN10	Average relative imporance	Normanlized relative importance (%)
*Model A (Output:PSY)*	EC	0.447	0.357	0.247	0.235	0.264	0.255	0.126	0.408	0.290	0.413	0.304	100.000
	PC	0.200	0.277	0.318	0.234	0.233	0.286	0.364	0.200	0.229	0.095	0.244	80.263
	HVA	0.207	0.168	0.246	0.250	0.266	0.252	0.273	0.208	0.284	0.043	0.220	72.368
	DFS	0.147	0.198	0.188	0.280	0.236	0.208	0.237	0.184	0.197	0.449	0.232	76.316
*Model B (Output:PHY)*	EC	0.362	0.344	0.278	0.243	0.388	0.382	0.290	0.351	0.397	0.295	0.333	100.000
	PC	0.212	0.254	0.267	0.240	0.224	0.184	0.223	0.231	0.226	0.198	0.226	67.868
	HVA	0.236	0.269	0.227	0.286	0.216	0.196	0.263	0.239	0.251	0.202	0.239	71.772
	DFS	0.190	0.134	0.227	0.232	0.172	0.238	0.224	0.179	0.127	0.305	0.203	60.961
*Model C (Output:JCI)*	EC	0.168	0.130	0.229	0.165	0.163	0.230	0.048	0.049	0.250	0.170	0.160	86.957
	PC	0.252	0.157	0.145	0.173	0.169	0.192	0.297	0.154	0.035	0.182	0.176	95.652
	HVA	0.122	0.174	0.149	0.088	0.169	0.105	0.142	0.106	0.156	0.099	0.131	71.196
	DFS	0.187	0.212	0.158	0.189	0.143	0.174	0.210	0.271	0.110	0.186	0.184	100.000
	PSY	0.186	0.203	0.169	0.204	0.225	0.141	0.139	0.126	0.120	0.168	0.168	91.304
	PHY	0.084	0.123	0.150	0.182	0.132	0.157	0.165	0.294	0.329	0.196	0.181	98.370

JCI, job crafting intention; PHY, physiological restoration; PSY, psychological restoration; DFS, distance from stressors; HVA, high visual authenticity; PC, personal compatibility; EC, experience continuity.

In Model C, where job crafting intention (JCI) serves as the output, the ranking of variable importance shifts. DFS shows the highest normalized importance (100.000%), followed by PHY (98.370%), PC (95.652%), PSY (91.304%), and EC (86.957%), while HVA, though comparatively lower, still reaches 71.196%. This suggests that when both restorative environmental perceptions and psychophysiological recovery variables are included, environmental characteristics associated with “being away from stress,” physiological recovery, and personal compatibility contribute most substantially to job crafting intention. Psychological recovery and experiential continuity play secondary roles, whereas high visual authenticity functions more as a supportive factor. Overall, experiential continuity consistently occupies a central position in predicting recovery outcomes (PSY and PHY), whereas distance from stressors and physiological recovery emerge as more prominent predictors when explaining job crafting intention.

### 5.3 Comparative analysis of predictive variable influence between SEM and ANN

To further explore the core roles of predictive variables in driving tourists' job crafting intention, this study compares the linear path coefficients derived from SEM with the nonlinear normalized importance rankings obtained from ANN (Table 13). First, ANN corrects potential ranking biases inherent in linear models. The SEM results indicate that Experience Continuity (EC) exerts the largest total effect (0.287), occupying a dominant position within the logical path structure. However, the sensitivity analysis of ANN reveals that, when nonlinear interactions among variables are taken into account, Degree of Being Away from Stressors (DFS; 100%) and Physiological Restoration (PHY; 98.37%) emerge as the most critical determinants of tourists' job crafting intention. This finding suggests that although experiential coherence enhances perception, the substantive triggers for proactive job crafting upon returning to the workplace are, in essence, the complete detachment from work-related stress and the restoration of physical energy.

**Table 13:** Comparison of SEM (linear overall effect) and ANN (nonlinear importance; with JCI as the output).

*Predictors*	SEM total effect	SEM ranking	ANN normalized importance (%)	ANN ranking	Identifying differentiators and adding value
*DFS*	0.230^**^	3	100.00	1	ANN identified “distance from stressors” as the strongest driver of intention
*PHY*	0.188^***^	5	98.37	2	ANN emphasized the threshold role of physical recovery in behavioral change
*PC*	0.249^**^	2	95.65	3	The findings from both studies are largely consistent, with both identifying it as a core driver
*PSY*	0.195^***^	4	91.30	4	The findings from both studies are largely consistent, indicating an important mediating role
*EC*	0.287^*^	1	86.96	5	It had the strongest overall effect in SEM, but its ranking declined in nonlinear integration
*HVA*	0.208^**^	6	71.20	6	Both studies consider it to play a foundational supporting role

^***^indicates *p* < 0.001;

^**^ indicates *p* < 0.01;

^*^ indicates *p* < 0.05; JCI, job crafting intention; PHY, physiological restoration; PSY, psychological restoration; DFS, distance from Stressors; HVA, high visual authenticity; PC, personal compatibility; EC, experience continuity.

Second, ANN quantifies the relative contribution weights of predictive variables, offering more precise prioritization guidance than SEM. While SEM determines statistical significance primarily through *p*-values, it is difficult to distinguish subtle differences among significant variables. By contrast, ANN employs normalized importance values to clearly demonstrate that DFS, PHY, and Personal Congruence (PC) constitute the “first-tier” drivers of intention (each exceeding 95% importance). This differentiated weight distribution provides a more operationally actionable reference for rural tourism destinations in formulating targeted intervention strategies.

## 6 Discussion and conclusions

### 6.1 Discussion

Grounded in the Stimulus–Organism–Response (S-O-R) theory, this study examines the relationship between restorative environments in rural tourism and respondents' job crafting intention. The SEM results indicate that environmental perception dimensions—namely, Degree of Being Away from Stressors, Visual Authenticity, Experience Continuity, and Personal Congruence—are all significantly and positively associated with job crafting intention. This finding supports the proposed linkage of “environmental perception → psychological/physiological restoration → job crafting intention.” It is consistent with restorative environment theory and prior research on nature exposure (60), which suggest that high-quality environmental experiences are statistically related to attentional recovery and emotional restoration. The present study extends the discussion of restorative experiences from the traditional focus on “well being” (61) to the domain of occupational psychology, specifically job crafting intention. The results suggest that rural tourism experiences are not only associated with individuals' physical and psychological recovery but may also relate to their willingness to reassess and proactively adjust their work approaches upon returning to the workplace.

By integrating SEM and ANN analytical strategies, this study observes noteworthy differences in the ranking of variable importance. SEM analysis reveals that the standardized path coefficient of psychological restoration on job crafting intention is slightly stronger than that of physiological restoration. However, ANN results indicate that the normalized importance of physiological restoration exceeds that of psychological restoration. Moreover, Experience Continuity (EC), which demonstrates the strongest total effect in SEM, drops to fifth place in the ANN nonlinear ranking, whereas Degree of Being Away from Stressors (DFS) rises to the top position. These discrepancies highlight the complementary value of the two statistical approaches. SEM, based on linear assumptions, reflects the average magnitude of effects among variables, suggesting that psychological relaxation is slightly more strongly associated with job intention under stable conditions. In contrast, ANN, as a nonlinear analytical tool, assigns greater importance to physiological restoration and detachment from stressors, potentially indicating that the recovery of physical functioning serves as a nonlinear driving force behind individuals' intention to initiate change. These differentiated findings suggest that destination managers should not rely solely on average linear effects but also consider the nonlinear importance rankings of factors in specific contexts, thereby providing tiered decision-making references for the precise optimization of tourism products.

From a theoretical perspective, the findings are consistent with restorative environment theory and related research on restorative experience scales. Previous studies have demonstrated that psychological restoration is closely linked to proactive behaviors, with positive emotions and a sense of meaning regarded as critical psychological resources that foster job crafting and creative performance (62, 63). Similarly, this study finds that the indirect effect through psychological restoration is slightly stronger overall than the pathway *via* physiological restoration, indicating that psychological resources may bear a more direct association with the formation of job crafting intention. Meanwhile, Experience Continuity (EC) emerges as the most important predictor of both psychological and physiological restoration. This finding echoes conclusions in leisure and vacation research suggesting that continuous, high-quality leisure experiences are more beneficial than fragmented breaks (61). Compared with brief or one-off visits, experiences characterized by contextual extension and spatial coherence are more likely to be associated with subsequent work-related attitudes.

Regarding moderating mechanisms, tourists' behavioral patterns exhibit significant moderating effects on the relationships between authenticity, personal congruence, experience continuity, and job crafting intention, but not on the pathway involving Degree of Being Away from Stressors. This suggests that merely being in an environment detached from stress does not necessarily correspond to significantly higher job crafting intention. Rather, more proactive and deeply engaged behavioral modes—such as sustained interaction with the environment, selecting activities closely aligned with personal interests, and actively participating in local cultural experiences—are more likely to strengthen the linkage between restorative perception and work-related attitudes. This finding aligns with leisure research emphasizing that active participation is more conducive to psychological growth (64) and enriches the restorative environment literature by incorporating the relatively underexplored dimension of individual agency. In summary, this study demonstrates a robust association between rural restorative environments and job crafting intention among working individuals. The findings suggest that rural tourism destinations should move beyond singular landscape development and instead construct in-depth “career-healing” scenarios encompassing detachment from stressors, experience continuity, and dual physical–psychological restoration. In the context of widespread modern work stress, rural spaces may thus be transformed into vital resource fields that promote psychological renewal and the revitalization of professional vitality within the labor force.

### 6.2 Conclusions

Grounded in the Stimulus–Organism–Response (S-O-R) framework, this study investigates the association between perceived restorative environmental qualities in rural tourism and job crafting intention among working professionals. Through an integrated analysis combining Structural Equation Modeling (SEM) and Artificial Neural Networks (ANN), the findings indicate that perceptions of the rural tourism environment—namely, Degree of Being Away from Stressors, Visual Authenticity, Experience Continuity, and Personal Congruence—are significantly and positively associated with respondents' job crafting intention. Psychological and physiological restoration function as parallel mediators within these relationships. It should be noted that both restoration status and job crafting in this study are measured based on respondents' subjective perceptions and self-reported intentions, reflecting associations among psychological resources rather than clinically validated health improvements or actual behavioral changes.

Furthermore, the complementary application of SEM and ANN reveals the complexity of the underlying influence mechanisms. While the linear SEM results suggest that the pathway through psychological restoration exhibits slightly stronger associations, the nonlinear ANN analysis highlights the prominent roles of physiological restoration and detachment from stressors in driving intention formation. This indicates that, upon fulfilling the foundational conditions of complete stress detachment and physical recovery, rural tourism experiences characterized by high continuity and alignment with personal traits demonstrate stronger statistical associations with individuals' propensity to proactively optimize their work patterns after returning to their jobs. Additionally, tourists' proactive behavioral patterns exert a positive moderating effect on the relationship between environmental perception and occupational intention.

Overall, this study underscores the potential value of rural tourism as a leisure-based intervention in relation to the regeneration of psychological resources and occupational behavioral intentions among working professionals. Its primary contribution lies at the intersection of occupational psychology and leisure behavior research, offering preliminary empirical evidence for understanding how tourism experiences may be associated with workplace psychological development.

### 6.3 Limitations and future directions

Although this study makes efforts in theoretical integration and methodological application, several limitations should be more explicitly acknowledged. First, the research relies on single-source self-reported questionnaire data, which may be subject to common method bias. Although certain procedural and statistical controls were implemented during the research design and analysis stages, reliance on a single data source may still inflate the observed correlations among variables. In addition, respondents were required to recall their rural tourism experiences and subjective restoration states, which introduces potential recall bias. Second, the sample primarily consists of employed individuals with relatively high educational attainment, medium-to-high income levels, and comparatively elevated self-reported stress. Such groups may be more attentive to restorative experiences and more inclined to reflect upon and adjust their work patterns, potentially amplifying the observed associations. Therefore, caution is warranted when generalizing the findings to populations with different occupational structures, income levels, or lower stress conditions. The external validity of the results should be further examined using more diverse samples.

Third, the cross-sectional design limits the ability to rule out reverse or reciprocal relationships among variables. For instance, individuals with stronger job crafting tendencies may be more likely to report higher levels of restoration or more positive environmental perceptions. Moreover, certain potentially influential variables—such as personality traits (e.g., proactive personality), perceived organizational support, job characteristics, or team climate—were not incorporated into the model. The omission of these variables may influence path estimations and introduce potential omitted variable bias. Fourth, at the methodological level, although the integration of SEM and ANN enhances predictive accuracy and reveals differences in variable importance rankings, ANN models inherently lack interpretive transparency, and their results depend on specific samples and parameter settings. Future research may consider employing more interpretable nonlinear models or adopting multi-method designs for cross-validation.

Future studies may extend this line of inquiry in several directions. First, longitudinal, experience sampling, or experimental designs could be employed to examine the temporal relationships between restorative experiences and work behaviors, while incorporating objective physiological or behavioral indicators to complement subjective measures. Second, the proposed model could be replicated across different regions, socioeconomic contexts, and cultural settings to enhance the generalizability of findings. Third, multilevel models may be constructed to situate individual-level restorative experiences within organizational and institutional contexts, thereby providing a more comprehensive understanding of the complex associations between leisure experiences and work-related attitudes.

## Data Availability

The original contributions presented in the study are included in the article/supplementary material, further inquiries can be directed to the corresponding author.
